# TEX101, a glycoprotein essential for sperm fertility, is required for stable expression of Ly6k on testicular germ cells

**DOI:** 10.1038/srep23616

**Published:** 2016-03-23

**Authors:** Shuichiro Endo, Hiroshi Yoshitake, Hiroki Tsukamoto, Hideyuki Matsuura, Ko Kato, Mayumi Sakuraba, Kenji Takamori, Hiroshi Fujiwara, Satoru Takeda, Yoshihiko Araki

**Affiliations:** 1 Institute for Environmental & Gender-specific Medicine, Juntendo University Graduate School of Medicine, Urayasu, Chiba 279-0021, Japan; 2 Department of Obstetrics & Gynecology, Juntendo University Graduate School of Medicine, Bunkyo-ku, Tokyo 113-8421, Japan; 3 Laboratory of Oncology, Pharmacy Practice and Sciences, Graduate School of Pharmaceutical Sciences, Tohoku University, Sendai, Miyagi 980-8578, Japan; 4 Laboratory of Applied Environmental Biology, Graduate School of Pharmaceutical Sciences, Osaka University, Suita, Osaka 565-0871, Japan; 5 Laboratory of Plant Metabolic Regulation, Graduate School of Biological Sciences, Nara Institute of Science and Technology, Ikoma, Nara 630-0192, Japan; 6 Department of Obstetrics & Gynecology, Kanazawa University Graduate School of Medical Science, Kanazawa, Ishikawa 920-8641, Japan

## Abstract

TEX101, a germ cell-specific glycosyl-phosphatidylinositol (GPI)-anchored glycoprotein, is associated with Ly6k during spermatogenesis in testis. Although both *Tex101*^*−/−*^ and *Ly6k*^*−/−*^ mice can produce morphologically intact spermatozoa, both knockout mice show an infertile phenotype due to a disorder of spermatozoa to migrate into the oviduct. Since Ly6k specifically interacts with TEX101, complex formation of TEX101/Ly6k appears to be potentially important for functional sperm production. This study evaluated the fate of Ly6k in the presence or absence of TEX101 to explore the molecular interaction of both GPI-anchored proteins in seminiferous tubules. The present study showed that: 1) Although *Ly6k* mRNA was detected, the protein was present at very low levels in mature testes of *Tex101*^*−/−*^ mice, 2) *Ly6k* mRNA level was within the normal range in *Tex101*^*−/−*^ mice, 3) *Ly6k* mRNA was translated into a polypeptide in the testes of *Tex101*^+/+^ and *Tex101*^*−/−*^ mice, and 4) TEX101, as well as Ly6k, are co-factors that affect to molecular expression. These results indicate that both TEX101 and Ly6k contribute to the post-translational counterpart protein expression at the cell membrane. This mechanism may be important in maintaining the production of fertile spermatozoa during spermatogenesis.

Mammalian spermatozoa are formed in the seminiferous tubules through a complex morphological/physiological alteration that includes meiosis and transformation^1^. These processes do not occur in cells of any other somatic organ. Therefore, it is generally believed that unique molecular mechanisms regulate these processes. In fact, many molecules are specifically expressed during germ cell formation^2^. However, the precise molecular mechanisms underlying germ cell formation, including functional maturations, remain unknown.

We previously identified TEX101, a glycoprotein that belongs to the Ly6/urokinase type plasminogen activator receptor-like protein (uPAR)(LU) superfamily, to be a germ-cell-specific molecule^3^,^4^. In males, TEX101 is found on the cell surface as a glycosyl-phosphatidylinositol (GPI)-anchored protein^5^. It first appears on the plasma membranes of prospermatogonia in immature seminiferous cords of the fetal testis during male gonadal development^4^. Although TEX101 is not expressed on spermatogonia, it reappears on meiotic cells and testicular spermatozoa after puberty^4^. TEX101 is then released from the surface of epididymal spermatozoa during transport through the caput epididymis^6^. Initially, TEX101 was thought to play an important role(s) in germ-cell formation through its cellular localization in the gonads during gametogenesis. However, *Tex101*^*−/−*^ mice produce morphologically intact spermatozoa, and the infertile phenotype is due to an inability of spermatozoa to migrate into the oviduct^7^.

In sexually mature testes, TEX101 is known to associate with several proteins, such as cellubrevin, annexin A2, and Ly6k^8^,^9^. Similar to TEX101, Ly6k is a member of the LU superfamily, which is characterized by the presence of conserved cysteine residues and a putative GPI-anchoring site in the amino acid sequence^10^. Since Ly6k specifically interacts with TEX101 during spermatogenesis^8^,^9^,^11^, and TEX101 is essential for production of fertile spermatozoa^7^, Ly6k may play an important role in the production of fertile spermatozoa. Regarding the biological function of Ly6k, no direct evidence is available. However, previous clinical studies demonstrated that LY6K (a human orthologue of murine Ly6k) is expressed in cancer cells of some patients with non-small cell lung carcinoma or oesophageal squamous cell carcinoma, as a new member of the cancer-testis antigens^12^,^13^. Since overexpression of LY6K was correlated with poor prognosis for patients with these carcinomas, this molecule is thought to possess biological functions such as cancer cell differentiation, proliferation, adhesion, and migration^12^. In addition, a soluble type of LY6K is occasionally detected in serum from patients with lung cancer or oesophageal cancer^12^, implying that measurement of the molecule in serum may be useful for diagnosis of carcinomas.

In the mouse testis, Ly6k was detected in all normal testicular fractions, which consist of extracellular (EC), water-soluble (WS), and Triton^TM^ X-100-soluble (TS) fractions using Western blot analysis^9^,^11^, showing that this molecule is located not only on the cell surface as a GPI-anchored protein, but also in the extracellular region of the testis as a soluble form. We have previously reported that TEX101 exists in EC regions of the testis^14^, similar to Ly6k. Many other GPI-anchored proteins, such as uPAR^15^, CD14^16^, CD16b^17^, CD55^18^, GPI-80^19^ are known to be released in soluble forms which may possess biological functions. For instance, soluble uPAR is known to induce smooth muscle cell activation and migration^20^. Although the biological functions of soluble types of both Ly6k and TEX101 remain unclear, the molecules may play a role in successful fertilization, similar to the GPI-anchoring forms.

Recently, a *Ly6k*^*−/−*^ mouse line was reported to show a phenotype similar to *Tex101*^*−/−*^ mice, with regard to expected reproductive ability^21^. Although previous preliminary studies have suggested that TEX101 influences the molecular expression of Ly6k and *vice versa*, the biosynthesis and molecular characteristics of Ly6k in seminiferous tubules remain unclear.

The aim of this study was to characterize the molecular nature of Ly6k more precisely during spermatogenesis. In particular, we focused on the fate of Ly6k as a TEX101-associate membrane protein in the presence and absence of TEX101.

## Results

### Validation of Ly6k transcription and translation in *Tex101*-deficient mice

**Figure 1:**
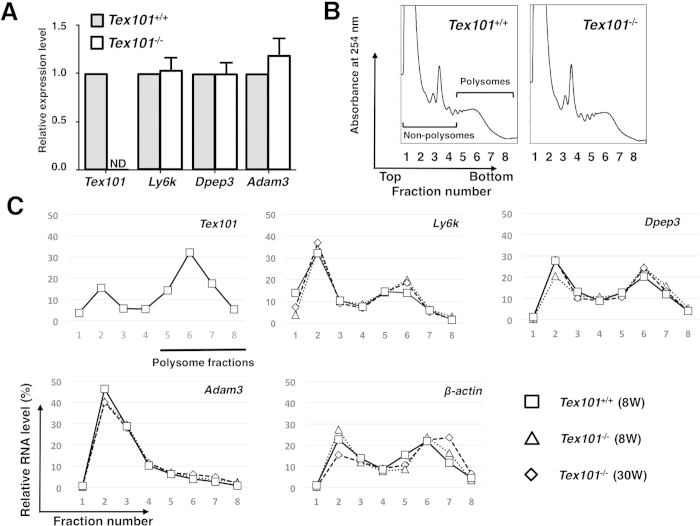
Differences of testicular gene expression between *Tex101*^+/+^ and *Tex101*^*−/−*^mice. Analysis of *Tex101*, *Ly6k*, *Dpep3*, and *Adam3* mRNA expression in the *Tex101*^*−/−*^ mice (**A**). Levels of these mRNA expressions in the testes of *Tex101*^+/+^ (n = 3) and *Tex101*^*−/−*^(n = 4) mice were measured using qRT-PCR with SYBR Green method. To obtain the ΔΔCt values for the calculation of fold increases, *β-actin* mRNA was used as a quantitative internal control. Average of each mRNA expression level in *Tex101*^+/+^ mouse testis was defined as “relative expression value = 1.0”. The data are indicated with SE. ND: not detectable. Polysome profiles (254 nm absorbance profile) of sucrose gradient sedimentation of cell extracts from the *Tex101*^+/+^ and *Tex101*^*−/−*^mice testes (**B**). Detergent-treated cell extracts were fractionated over a 15–60% sucrose density gradient into eight fractions of equal volume. Expression levels of testicular *Tex101*, *Ly6k*, *Dpep3*, *Adam3*, and *β-actin* mRNAs from the 8-week *Tex101*^+/+^ and 8- or 30-week-old *Tex101*^*−/−*^mice in each fraction of the sucrose gradients (**C**). Each fraction number corresponds to that in the absorbance profile shown in (**B**). Data are graphically represented as ratio of transcripts from a typical experiment, present in each fraction to the sum of all fractions. As an external standard, *r-luc* mRNA was used.

A recent study used Western blot analysis to show that the expression of Ly6k was absent in the testes of *Tex101*^*−/−*^21. To clarify the mechanism in the testes derived from the TEX101-null mouse, we first investigated whether disruption of *Tex101* affected *Ly6k* transcription in the testis. Quantitative real-time (qRT)-PCR analysis revealed that *Ly6k* mRNA expression levels in the testes were nearly identical in *Tex101*^*−/−*^ and control mice (Fig. 1A). Similar to *Ly6k*, levels of *dipeptidase 3* (*Dpep3*) and *Adam3*, both molecules related to TEX101 in the testis^7^,^22^, were not significantly different in the presence or absence of TEX101 (Fig. 1A).

As the next step, the mRNA expression levels of TEX101-related molecules in the *Tex101*^*−/−*^ mouse testis were further confirmed using a DNA microarray analysis. As expected, the *Tex101* mRNA level was downregulated in the *Tex101*^*−/−*^ mouse testis (Supplementary Table S1). Other genes expressed in the testis, such as *Ly6k*, *Dpep3*, *angiotensin I converting enzyme* (*Ace*), *Adam3*, and *sperm acrosome associated 4* (*Spaca4*) did not show significant changes in expression between *Tex101*^*−/−*^ and *Tex101*^+/+^ mice (Supplementary Table S1). We next evaluated the translation state in the testes of *Tex101*^*−/−*^ and *Tex101*^+/+^ mice. In this examination, qRT-PCR analysis combined with a polysome fractionation assay^23^,^24^ was performed. When detergent-treated testicular extracts were fractionated using sucrose density gradient centrifugation, fractions 1–4 and 5–8 corresponded to non-polysomal and polysomal regions, respectively (Fig. 1B). There was no significant difference in the polysomal and non-polysomal profiles between *Tex101*^+/+^ and *Tex101*^*−/−*^ mice (Fig. 1B). These data suggested that disruption of the *Tex101* gene does not widely affect the translation of other genes.

Attempts were then made to analyze the expression levels of various testicular genes in each fraction using qRT-PCR. In the wild-type mouse, *Tex101* mRNA was distributed in both non-polysomal and polysomal fractions (Fig. 1C). A peak level was observed in polysome fractions (Fig. 1C), implying that the *Tex101* translation step in the testis is intact. *Ly6k* mRNA was similarly distributed to *Tex101* in the polysomal fractions obtained from *Tex101*^+/+^ and *Tex101*^*−/−*^ mice. The *Ly6k* expression levels in each fraction were also nearly identical in these mice (Fig. 1C). The mRNA expression levels of other testicular molecules, such as *Dpep3, Adam3*, and the housekeeping gene (*β-actin*) in each fraction were not significantly different between *Tex101*^+/+^ and *Tex101*^*−/−*^ mice (Fig. 1C).

### Expression pattern of Ly6k with/without TEX101 in the testicular seminiferous tubules

**Figure 2:**
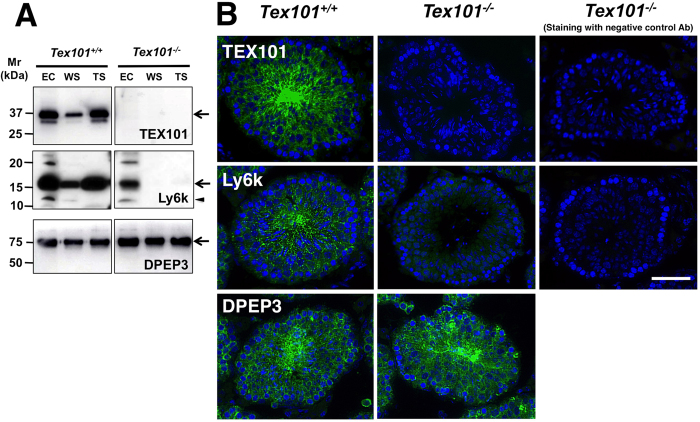
Effects of *Tex101* gene disruption on the expression of testicular proteins. Western blot analysis (**A**). The EC, WS, and TS fractions of testicular proteins from *Tex101*^+/+^ or *Tex101*^*−/−*^ mice were applied to each lane of a 15% SDS-PAGE gel, separated under non-reducing (TEX101) or reducing (Ly6k and DPEP3) conditions. After electroblotting, the immunoreactivity of the Abs against TEX101, DPEP3, or Ly6k was monitored with an ECL detection system. Mr: molecular mass. Arrows indicate the main immunoreactive bands detected with each specific Ab. A non-membrane-binding form of Ly6k at the apparent molecular mass of 12-kDa is indicated by an arrow head. Immunofluorescent analysis (**B**). PFA-fixed frozen sections of the *Tex101*^+/+^ or *Tex101*^*−/−*^ mouse testis were incubated with the anti-TEX101, DPEP3, or Ly6k Abs, or isotype-matched NC Abs corresponding to each primary Ab (green), and following nuclear staining with DAPI (blue). All seminiferous tubules shown are stage VIII based on nuclear staining. Bars: 50 μm.

Experimental results obtained from testicular polysome analyses (Fig. 1C) suggested that the downregulation of Ly6k protein within the *Tex101*^*−/−*^ mouse testis may be due to disorder(s) after polypeptide synthesis. To clarify this possibility, we investigated the subcellular localization of Ly6k in the testis. Previous studies have reported that both Ly6k and TEX101 are localized in all normal testicular fractions, which consist of EC, WS, and TS fractions using Western blot analysis^9^,^14^. Confirmative experiments using *Tex101*^*−/−*^ mouse testis by Western blot analysis did not detect TEX101 in any fraction, as expected (Fig. 2A, right top panel). In contrast, Ly6k was not clearly observed in WS and TS fractions from *Tex101*^*−/−*^ mice. Although Ly6k was detected in the EC fraction in the *Tex101*^*−/−*^ mouse, the expression level appeared to be decreased compared to the wild-type mice (Fig. 2A, right middle panel). An additional band at the apparent molecular mass of 12-kDa (indicated by an arrow head, Fig. 2A, right middle panel) detected with anti-Ly6k pAb used in this experiment is a non-membrane-binding form of Ly6k^11^. DPEP3, a testicular GPI-anchored protein^22^, was detected in all testicular fractions derived from *Tex101*^*−/−*^ mice, as well as *Tex10*^+/+^ mice (Fig. 2A, bottom panel).

Immunofluorescent experiments demonstrated that TEX101 and Ly6k were present in the testicular seminiferous tubules from a wild-type mouse, in spermatocytes, spermatids, and testicular spermatozoa (Fig. 2B, upper and middle left panels), as shown in our previous studies^3^,^9^,^11^. On the other hand, Ly6k was slightly expressed in the seminiferous tubules of *Tex101*^*−/−*^ mouse testis (Fig. 2B, center panel). Expression patterns of DPEP3 in the testis were not different between *Tex101*^+/+^ and *Tex101*^*−/−*^ mice (Fig. 2B, bottom left and middle panels).

### Intracellular localization of Ly6k and TEX101 in the testes

**Figure 3:**
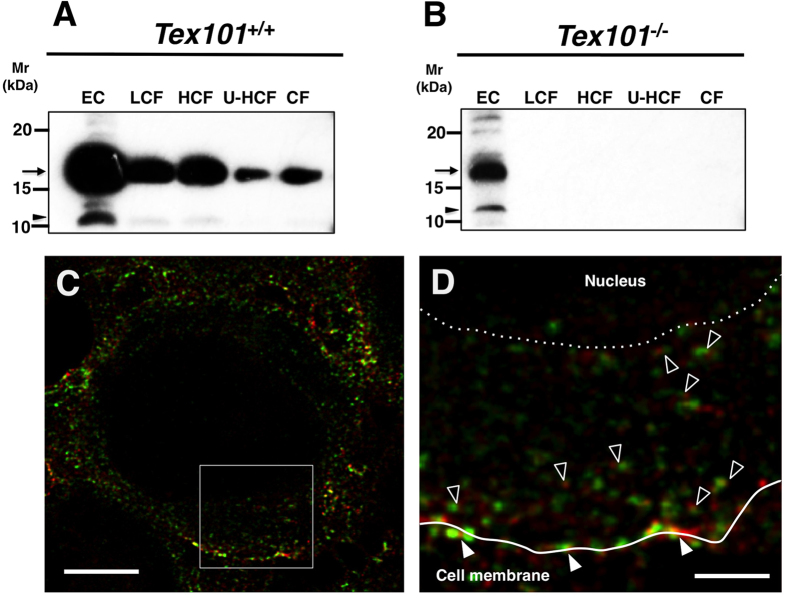
Subcellular distributions of Ly6k in the testes derived from *TEX101*^+/+^ and *Tex101*^*−/−*^mice. Western blot analyses of extracellular and testicular-cell fractions (**A,B**). The testicular-cell fractions prepared with the differential centrifugation method as described “Materials and Methods” section. The samples were adjusted to same cell numbers in equal volume (10 μl), applied to each lane of 15% SDS-PAGE gels, electroblotted onto a PVDF membranes, and then probed with the anti-Ly6k pAb. EC: extracellular fraction; LCF: fraction by low-speed centrifugation; HCF: fraction by high-speed centrifugation; U-HCF: fraction by ultrahigh-speed centrifugation; and CF: cytosol fraction. Apparent molecular mass (Mr) of Ly6k is indicated by an arrowhead at the left margin of each panel. Immunofluorescent analyses of TEX101 and Ly6k within the seminiferous tubules of *Tex101*^+/+^ mouse (**C,D**). Testicular cryosections (3 μm in thickness) were incubated with the primary antibodies at 4 °C overnight and subsequently with Alexa Fluor 488 or Alexa Fluor 555-conjugated secondary Abs. The specimens were observed by a TCS SP8 STED 3X confocal laser microscopy. The localizations of TEX101 and Ly6k are indicated as red and green, respectively. An overlay image is showed in C. Panel (**D**) shows a higher magnified image of the boxed region in panel (**C**). Co-localization of TEX101 and Ly6k on the plasma membranes and in cytoplasmic regions is indicated with closed and open arrowheads, respectively. A solid line in D indicates cell surface of a TGC, and a dotted line represent a border between cytoplasm and nucleus of the cell. Bars: 5 μm (**C**); 1 μm (**D**).

To further investigate the lower Ly6k expression in the testes of *Tex101*^*−/−*^ mice, the subcellular localization of Ly6k was examined. Testicular cell extracts were divided into the following five fractions (for details, see “Materials and Methods”): EC, a fraction obtained by low-speed centrifugation (LCF) containing nuclear membrane, a fraction obtained by high-speed centrifugation (HCF) containing lysosomal and mitochondrial proteins, a fraction obtained by ultrahigh-speed centrifugation (U-HCF) containing other membrane proteins, including vesicle components, and a cytosol fraction (CF). Western blot analysis revealed the presence of Ly6k in all fractions from wild-type mice (Fig. 3A). In *Tex101*^*−/−*^ mouse testis, Ly6k was detected only in the EC fraction, not in any of the other intracellular fractions (Fig. 3B). It was reported previously that Ly6k is present in spermatocytes, spermatids, and testicular spermatozoa in the testis of sexually mature mice, and is co-localized with TEX101 on the plasma membranes of these cells using conventional confocal microscopy^9^. To examine whether Ly6k was also expressed and co-localized with TEX101 in the intracellular region of testicular germ cells (TGCs), a more precise immunofluorescence study was performed using a stimulated emission depletion (STED) confocal laser microscope. In the wild-type mouse, TEX101 was detected on the plasma membrane of spermatocytes, spermatids, and testicular spermatozoa of the testis, and exhibited a spotted distribution in the cytoplasmic regions of spermatocytes and spermatids, as reported previously^3^. The expression pattern of Ly6k was similar to that of TEX101, and the majority of Ly6k staining overlapped with that of TEX101 on the plasma membrane and in the cytoplasmic region of TGCs (Fig. 3C,D).

### Effects of *Tex101*/*Ly6k* siRNAs on their expression in a stable transfectant cell line

**Figure 4:**
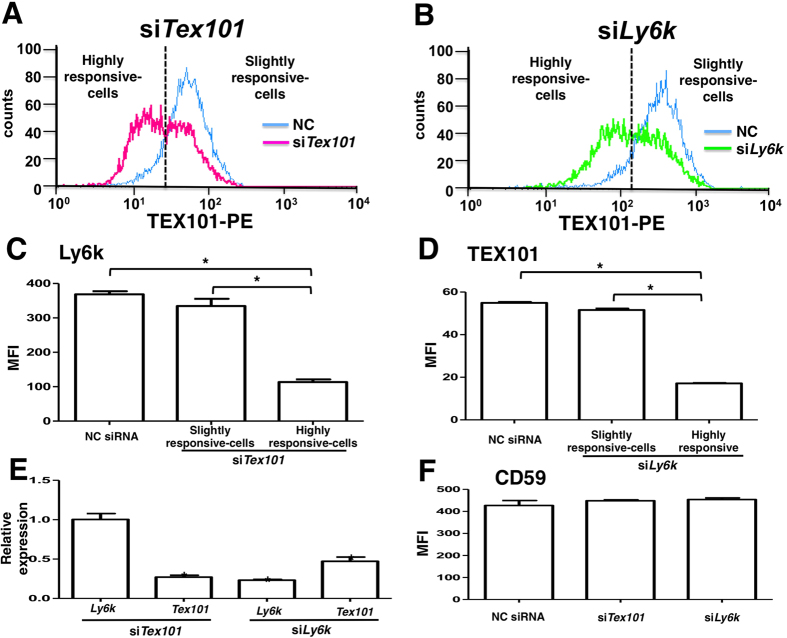
Effects of siRNAs against *Tex101* and *Ly6k* genes on the expression of those molecules in 293mTx/ly6kκB cell line. After transfected with each siRNA and sequentially incubated for 48 h in a 5% CO_2_ humidified atmosphere at 37 °C, the 293mTx/ly6kκB cells were harvested. Expression levels of TEX101 and Ly6k on the transfected cell surfaces were measured by flow cytometry. Histograms of the cells transfected either *Tex101* (**A**), or *Ly6k* (**B**) siRNA. Blue lines represent the histogram of NC siRNA transfected cells. The left sides peak of dotted lines contain cells that are highly responsive for si*Tex101* (**A**) or si*Ly6k* (**B**), respectively. The other side peaks contain cells that are slightly response for each siRNA. Expression of Ly6k (**C**) and TEX101 (**D**) in the 293mTx/ly6kκB cells transfected with NC, *Tex101*, or *Ly6k* siRNA. The data are expressed as the mean fluorescence intensity (MFI) with SE measured by flow cytometry (*n* = 3). *p < 0.05. Changes in mRNA expression levels of *Ly6k* and *Tex101* in the *TEX101*- or *Ly6k* siRNA-transfected 293mTx/ly6kκB cells (**E**). The mRNA expression levels were measured by the qRT-PCR analysis with SYBR Green method. Relative expression values were normalized with the *Rps18* expression and the normalized values of each mRNA expression in the transfected cells with NC siRNA are defined as “1.0”. Values are shown as means with SE (*n* = 3). *p < 0.05 compared to the control (NC siRNA transfection). Effects of *Tex101* and *Ly6k* siRNAs on CD59 expression (**F**). After 293mTx/ly6kκB cells were transfected with NC siRNA, *siTex101*, or *siLy6k* and then incubated, CD59 expression levels were analyzed by flow cytometry. The data shown are the MFI with SE (*n* = 3).

From the data presented in Figs 1, 2, 3, we hypothesized that Ly6k protein expressed on the plasma membrane and in the intracellular region of germ cells would be unstable in the absence of TEX101. To investigate whether TEX101 is required for stable expression of Ly6k on the cell surface, flow cytometry analysis of a TEX101/Ly6k-stable transfectant (293mTx/ly6kκB) was performed using siRNAs to these molecules. We initially confirmed that the *Tex101* siRNA transfection system used in our experiments reduced the expression level of TEX101 protein (Fig. 4A). When 293mTx/ly6kκB cells were transfected with *Tex101* siRNA, the cell group that displayed the greatest reduction in TEX101 level also exhibited a significant reduction in the expression level of Ly6k (Fig. 4C). One possibility considered was that direct binding of *Tex101* siRNA to *Ly6k* mRNA might downregulate Ly6k. To rule out this possibility, *Ly6k* mRNA levels in *Tex101* siRNA-transfected cells were measured using qRT-PCR. Transfection of *Tex101* siRNA had no effect on the *Ly6k* mRNA level (Fig. 4E).

Next, TEX101 expression in 293mTx/ly6kκB cells transfected with *Ly6k* siRNA was investigated. After examining the effect of the *Ly6k* siRNA on the Ly6k protein levels (Fig. 4B), the TEX101 expression in transfected cells was investigated. In the cell group that showed the greatest response to *Ly6k* siRNA, the TEX101 protein level was significantly downregulated (Fig. 4D). *Ly6k* siRNA also decreased *Tex101* mRNA expression (Fig. 4E), suggesting that the downregulation of TEX101 protein may be dependent on off-target effects of the *Ly6k* siRNA. However, neither *Tex101* nor *Ly6k* siRNA affected expression of CD59, an endogenous GPI-anchored protein in HEK293 cells (Fig. 4F).

## Discussion

On the surface of mice TGCs, we initially reported the association of GPI-anchored proteins, Ly6k and TEX101^8^,^9^. We further characterized Ly6k in terms of its expression during testicular development, using specific antibodies (Abs)^9^,^11^. According to these pioneering studies, as well as phenotypic data derived from a recent gene disruption study^21^, Ly6k is thought to be one of the important molecules for the production of spermatozoa with fertilizing ability *in vivo*. In the present study, we demonstrated, for the first time, that both Ly6k and TEX101 contribute to the molecular expression to each other in protein level during biosynthesis in TGCs.

Although a previous report has shown that Ly6k is absent from whole testicular lysates from *Tex101*^*−/−*^ mice^21^, more precise Western blot and immunohistochemical analyses using a Ly6k-specific Ab revealed marked Ly6k expression only in the testicular EC fraction, but not in the other fractions from sexually mature *Tex101*^*−/−*^ mice (Fig. 2A, right middle panel). However, the total quantity of Ly6k appears to be reduced in the absence of TEX101 when compared to the testis of wild-type mice (Fig. 2). Since Ly6k should be predominantly distributed in the testicular EC and TS fractions in wild-type mice^9^ (Fig. 2), these results suggest that Ly6k expression is affected (including its subcellular localization) in the presence or absence of TEX101.

Microarray and qRT-PCR data (Fig. 1A, Supplementary Table S1) suggested that the low expression of testicular Ly6k in the absence of TEX101 presumably originated from abnormalities in the posttranscriptional step of Ly6k-biosynthesis. As in the case of cellubrevin^8^, Ly6k and TEX101 are morphologically co-localized within the cytoplasm of TGCs, *i.e.*, data obtained from STED microscopic analyses (Fig. 3C,D) were indicative of molecular formation of Ly6k/TEX101 complexes not only on the plasma membrane but also in the cytoplasm, including intracellular vesicles of TGCs in wild-type mice. It seems that Ly6k/TEX101 complex formation is completed in the transport vesicles of wild-type mice.

Regarding GPI-anchored proteins, several molecules, such as post-GPI attachment proteins related to posttranslational modification during biosynthesis have been identified (for review, see Kinoshita^25^). Various disorders arise from posttranslational modification of GPI-moieties on GPI-anchored proteins, causing their abnormal selective transportation from the endoplasmic reticulum (ER) to the Golgi apparatus^26^, or disturbed lipid raft formation at the cell membrane^27^,^28^. From this point of view, reduction of Ly6k expression in the testes of *Tex101*^*−/−*^ mice may not be the case since a normal expression level of testicular GPI-anchored protein, DPEP3, was maintained in the gene-disrupted animals (Fig. 2).

A recent report concerning TEX101 expression in the testis of the *Ly6k*^*−/−*^ mouse also showed a significant reduction in TEX101^21^, suggesting that TEX101/Ly6k are complementary molecules and that disruption of the gene encoding one will affect the expression of the other. It should be noted that data obtained from polysome analyses of the testes of *Tex101*^+/+^/*Tex101*^*−/−*^ mice showed that there was almost no difference in the distribution of testicular genes in the polysome fractions. These results strongly suggest that disruption of the *Tex101* gene does not affect the transcriptional or translational activities of *Ly6k*, *Dpep3*, or *Adam3* within the testis. Taken together, these results demonstrate that Ly6k biosynthesis is completed at the translational step, and that low testicular expression of Ly6k in the absence of TEX101 is not due to a disorder of common posttranslational modifications of GPI-anchored proteins.

Gene knockdown of either *Tex101* or *Ly6k* by siRNAs transfected into a stable TEX101/Ly6k double positive cell line showed that both TEX101 and Ly6k appeared to contribute to the stability of the TEX101/Ly6k protein complex (Fig. 4C,D), although off-target effects of *Ly6k* siRNA on *Tex101* expression cannot be completely ruled out (Fig. 4E). However, expression of CD59, a GPI-anchored protein in HEK293 cells, did not change significantly after transfection with siRNA against *Tex101* or *Ly6k* (Fig.4F), suggesting that both TEX101 and Ly6k contribute to the stability of the TEX101/Ly6k protein complex on the plasma membrane.

**Figure 5:**
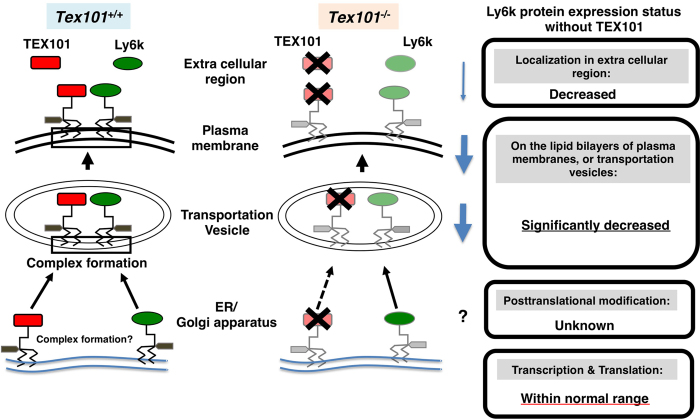
Molecular status of Ly6k with/without TEX101 in the TGCs. The left diagram indicates TEX101/Ly6k complex formation of wild-type (*Tex101*^+/+^) mouse. After translation, GPI remodeling of these molecules is completed from ER to Golgi apparatus, then these molecules are expressed as TEX101/Ly6k complex (represented by black square) on lipid bilayers including transportation vesicle and plasma membrane. In addition, (a part of) both TEX101 and Ly6k are released into extracellular space. In TEX101-null TGCs (the right diagram), Ly6k expression is drastically decreased. Black cross marks indicate the disruption of the molecules. The potential status of Ly6k protein expression without TEX101 is boxed.

Based on these results, the molecular status of Ly6k in TGCs in the presence and absence of TEX101 is summarized (Fig. 5). It is likely that Ly6k protein production (transcription and translation) is completed in the ER of TGC in *Tex101*^*−/−*^ mice. After translation, Ly6k does not maintain its appropriate position for unknown reason(s) on the membrane, presumably from Golgi apparatus to plasma membrane, resulting in a significant decrease in Ly6k in these lipid bilayers.

To date, several GPI-anchored proteins have been identified in TGCs. Among these, TEX101 and Ly6k are known to be essential for the production of functionally intact sperm^7^,^21^. Although the phenotypes of both TEX101- and Ly6k-null mice resemble those of several gene-disrupted mouse strains including *Adam3*^*−/−*^ mice^7^,^21^,^29^,^30^,^31^, the precise molecular mechanisms causing infertility of these spermatozoa remain unknown.

We suggest taking caution before making scientific conclusions as to the molecular significance of ADAM3-related molecule(s) at the protein level. For example, subcellular localization of Ly6k within TGCs is controversial^9^,^21^, presumably depending on the sample preparation as well as analytical methods used. Indeed, we previously reported molecular diversity of TEX101 in TGCs using various original monoclonal Abs (mAbs)^14^. Therefore, similar results may be observed for other ADAM3-related molecules including Ly6k. In addition, no appropriate molecular probe for ADAM3 detection at protein level (especially for morphological analyses of the molecule) is available which has limited our understanding of the molecular relationship between ADAM3 and its related molecules. It should be noted that experimental data obtained from polysome analysis suggest that *Adam3* message is expressed, but detectable translation activity is not observed in the adult testes (Fig. 1C), suggesting that the majority of ADAM3-biosynthesis does not likely occur in the adult testis. In the epididymal mature sperm, it is still unclear how ADAM3 is produced and where its production occurs. To address these questions, there may be limitations in using a gene disruption approach. Further studies will be required to elucidate the exact molecular mechanism(s) needed to produce functionally intact spermatozoa *in vivo*.

In summary, this study demonstrated that both TEX101 and Ly6k contribute mutually for their protein expression in the TGCs, suggesting that the expression-balance of these two ADAM3-related testicular GPI-anchored proteins is essential for fertile sperm formation. Accordingly, a similar or unexpected molecular status may be observed during the production of spermatozoa in the other ADAM3-related molecules^31^ that have phenotypes similar to those in *Adam3*^*−/−*^ mice.

## Materials and Methods

### Animals

The *Tex101*-disrupted (*Tex101*^*−/−*^) mice were established as reported previously^7^. They were maintained and bred at the animal facilities of the Juntendo University under 12L: 12D conditions, and given free access to food and water. Animal experimental protocols were approved by the ethical committee for laboratory animals of Juntendo University (the approved # 260036). All procedures were conducted according to the guide for care and use of laboratory animals, Juntendo University.

### Abs

TES101; (a mouse anti-TEX101 mAb: IgG1)^3^, mk34; (a rat anti-Ly6k mAb: IgG2a)^11^, a rabbit anti-Ly6k polyclonal Ab (pAb)^9^, and a rabbit anti-mouse DEPE3 pAb^22^ were produced and purified as reported previously. An anti-mouse TEX101 mAb, mTX5.2 (rat IgG2a) was generated essentially according to the method as described previously^11^. Mouse anti-human GPI-80 mAb (3H9, IgG1)^32^ was used as an isotype control for TES101 mAb. For some experiments, primary Abs were directly labeled with fluorescence dye using Alexa Fluor^®^ 488 Ab Labeling Kit (Molecular Probes, Eugene, OR, USA). Other Abs used in this study were purchased from the following companies: Alexa Fluor 488-conjugated goat anti-rabbit, rat or mouse IgG pAb, Alexa Fluor 594-conjugated goat anti-mouse IgG pAb, Alexa Fluor 555-conjugated goat anti-mouse IgG pAb, and R-phycoerythrin (RPE)-conjugated mouse anti-human CD59 mAb (Molecular Probes); normal control mouse IgG, rat IgG2a, and rabbit Igs, horseradish peroxidase (HRP)-conjugated goat anti-rabbit or rat Ig pAb and rabbit anti-mouse IgG pAb, RPE-conjugated rabbit anti-mouse Ig pAb (DAKO, Glostrup, Denmark).

### Preparation of mouse testicular extracts

Mouse testicular extracts separated into three fractions classified, as EC, WS, and TS, were prepared according to the methods as described before^14^. Briefly, a testis from sexually mature mouse was homogenized with a glass homogenizer 20 strokes in 9 volumes of phosphate-buffered saline (PBS; pH7.4) containing 1 × EDTA-free complete inhibitor cocktail (Roche Diagnostics GmbH, Penzberg, Germany) (buffer A) and then filtered through nylon mesh. After centrifugation at 200 × g for 5 min, the supernatant was collected and used as the EC fraction. The precipitate was washed with PBS, then resuspended in equal volume of buffer A and homogenized by Sonifier ultrasound homogenizer (Branson, Danbury, CT, USA) for 10 s six times. After ultracentrifugation at 114,000 × g for 30 min, the supernatant was collected and used as the WS fraction. The precipitate washed with PBS was resuspended in buffer A containing 1% Triton X-100. Following a 20-min incubation, the suspension was ultracentrifuged at 114,000 × g for 30 min, and resultant supernatant used as the TS fraction. All subsequent steps were performed at 4 °C. All samples were stored at −80 °C until use.

For cellular organelle fractionation, testicular-cell suspension was separated by differential centrifugation of homogenates according to the method essentially described by Hogeboom^33^ with slight modifications as follows: a mouse testis was homogenized with a glass homogenizer in 10 mM Tris-HCl buffer (pH7.4) containing 0.25 M sucrose, 1 mM EDTA, and 1 × Complete inhibitor cocktail (buffer B) and then centrifuged at 200 × g for 5 min, the supernatant was collected and used as the EC fraction. The pellet was washed with buffer B and homogenized with a Teflon^®^ homogenizer for 1 min. After the suspending solution was centrifuged at 1,000 × g for 10 min, the pellet was collected as LCF. The supernatant was further centrifuged at 15,000 × g for 15 min, and then the pellet was collected as HCF. After the supernatant was re-centrifuged at 100,000 × g for 60 min, the pellet and final supernatant were collected as U-HCF and CF, respectively. The pellets collected at each point were washed with buffer B, then resuspended in buffer B containing 1% Triton X-100. After incubation for 20 min on ice, the suspensions were centrifuged at 114,000 × g for 30 min. The resultant supernatants were used as samples for Western blotting analysis. Each testicular fraction was divided into small aliquots, and kept frozen (−80 °C) until use.

### Western blot analysis

The mouse testicular extracts were separated by standard SDS-PAGE system under reducing or non-reducing conditions^3^. The molecular mass of the antigen reactive with the primary Abs was determined by enzyme immunostaining of the protein after blotting to a polyvinylidene difluoride membrane (Millipore, Bedford, MA, USA) from the SDS-PAGE gel as described previously^3^,^8^.

### Immunohistochemistry

Sections (3–6 μm in thickness) of 4% paraformaldehyde (PFA)-fixed mouse testis were prepared as described previously^9^. The specimens were incubated with the primary Abs, labeled with/without florescent dye at 4 °C overnight. In the case of indirect immunostaining, the specimens were further treated with Alexa Fluor 488, 555, or 594-conjugated secondary Abs for 1 h at room temperature. In some experiments, Alexa Fluor 488-labeled primary Abs were used for antigen detection. The specimens were sequentially counterstained with 4,6-diamidino-2-phenylindole dihydrochloride (DAPI) (Invitrogen) and mounted in the ProLong Gold antifade reagent (Invitrogen). The immunostained tissues were observed and analyzed by a BIOREVO BZ-9000 microscope system (KEYENCE, Osaka, Japan) or a STED confocal laser microscope (TCS SP8 STED 3X; Leica Microsystems CMS GmbH, Mannheim, Germany).

### RNA isolation

Total RNA was isolated from each mouse organ using ISOGEN II (NIPPON GENE, Toyama, Japan) according to the manufacture’s protocol. Contaminated DNA was degraded using the DNA-free Kit (Life Technologies, Carlsbad, CA, USA). The quantity and quality of total RNAs were determined by a spectrophotometer. The concentration of total RNA was regulated at 700–1,200 ng/μl with RNase free water (RFW).

### Polysome fractionation assay

Polysomes were fractionated by sucrose density gradient centrifugation as described previously^23^,^24^ with minor modifications as follows: Testes from sexually mature mice were removed and pulverized in liquid nitrogen with a mortar and pestle. The resulting frozen powder was homogenized in 5 volumes of 200 mM Tris-HCl buffer (pH 8.5) containing 50 mM KCl, 25 mM MgCl_2_, 2 mM EGTA, 100 μg/ml heparin, 2% polyoxyethylene 10-tridecyl ether, and 1% sodium deoxycholate. Then the sample was centrifuged at 15,000 × g for 10 min at 4 °C. Aliquots of the supernatant were layered on 4.6 ml of a 15–60% sucrose density gradient in 50 mM Tris-HCl buffer (pH 8.5) containing 25 mM KCl and 10 mM MgCl_2_, and centrifuged at 287,000 × g for 50 min at 4 °C. The gradient was collected from the bottom and fractionated into eight fractions of approximately 650 μl each using a peristaltic pump with simultaneous recording of absorbance probes at 254 nm using a UV monitor (GE Healthcare Buckinghamshire, UK). Guanidine hydrochloride at a final concentration of 5.5 M, together with 5 ng of *in vitro* synthesized capped, polyadenylated *Renilla luciferase* (*r-luc*) mRNA^24^, was preliminarily added to the each collection tube. The *r-luc* mRNA was used as an external control to correct for varying efficiencies of subsequent RNA isolation and qRT-PCR processes. The RNA was precipitated from each fraction by the addition of an equal volume of ethanol, then incubated for overnight at −20 °C. After centrifugation at 7,380 × g for 45 min, the resulting precipitate was washed with 80% ethanol. Subsequent RNA purification was performed using the RNeasy kit (Qiagen, Hilden, Germany) with on-column DNase 1 treatment according to the manufacturer’s instructions. The RNA was eluted by 30 μl of RFW. Equal volumes of the RNA solution from each fraction were subjected to qRT-PCR analysis.

### Analysis by qRT-PCR system

To synthesize cDNA, total RNA (100–620 ng) or RNA isolated from each gradient fraction (200 ng) was reverse transcripted using the PrimeScript RT regent Kit (Takara, Shiga, Japan). The qRT-PCR analysis was performed using gene-specific primer sets (Supplementary Table S2), which were designed using Primer 3 Plus software^34^ and were purchased from Life Technologies. SYBR^®^ Green method for qRT-PCR was carried out using SYBR^®^ Premix Ex Taq^TM^ (TaKaRa) with the ABI PRISM^®^ 7900 HT Sequence Detection System (Life Technologies) under the following conditions: an initial denature step at 95 °C for 30 s, 40 cycles at 95 °C for 5 s and 60 °C for 34 s; dissociation stage at 95 °C for 15 s, 60 °C for 60 s, and 95 °C for 15 s. The qRT-PCR data were analyzed using the 2^−ΔΔCt^ method.

### Microarray analysis

The total RNA samples (2μg) were reverse transcribed to cDNA, which was further transcribed into amino allyl-modified aRNA using the Amino Allyl MessageAmp II aRNA Amplification Kit (Life Technologies). The resulting aRNA was labeled with Cy3 and Cy5 dyes (GE Healthcare), then the labeled samples were loaded onto a 3D-Gene Mouse Oligo chip 24k ver.1.1 (TORAY, Tokyo, Japan), and then incubated for 16 h at 37 °C. The signal intensity data were quantified from hybridization images using a GenePix 4400a (Molecular Devices, Sunnyvale, CA, USA) and analyzed using GenePix Pro7 software (Molecular Devices). Any genes displaying signal intensities less than or equal to 10 both Cy5 and Cy3 were omitted from the data list. The resulting values were normalized using the global median normalization. Furthermore, the genes showing signal intensities less than or equal to 100 were omitted in order to define the credibility of the dataset. Finally, the Cy5/Cy3 ratio was calculated for all available genes. Whole analyses were carried out using the Excel (Microsoft, Redmond, WA, USA) and Avadis software programs (Strand Life Sciences, Bangalore, India).

### Establishment of stable transfectant cells

Stable transfectant HEK293κB cells expressing mouse Ly6k (293mly6kκB) were previously established^11^. A HEK293κB cell-line co-expressing TEX101/Ly6k was established by the co-transfection of pCD-TEX101^5^ and mly6k/pCAGGS1^11^ with a pBabePuro vector (Cell Biolabs, San Diego, CA, USA) and following cultivation with puromycin. The TEX101/Ly6k-double positive stable cells were screened by flow cytometry with the mTX5.2 and mk34 mAbs. A stable transfectant cell-line expressed both TEX101 and Ly6k, termed 293mTx/ly6κB, was successfully established.

### Transfection of short interfering RNA (siRNA) and flow cytometry analysis

The 293mTx/ly6κB cells were diluted with the incubation medium containing 10% FCS and seeded onto a 12-well microplate (IWAKI Glass, Tokyo, Japan). After 48-h incubation at 37 °C in 5% CO_2_ in air, the incubation medium were changed to the same medium containing with 100 nM of siGENOME GAPD Control, non-targeting siRNA#1, mouse *Tex101*, or mouse *Ly6k* siRNA-SMART pool (GE Dharmacon, Lafayette, IN, USA) and 2 μl of DharmaFECT1 Transfection Reagent (GE Dharmacon), and then incubated for 48 h at 37 °C in 5% CO_2_ in air. After incubation, the cells were labeled with the primary and secondary Abs, following nucleus staining with propidium iodide. The expression levels of molecules on the cell surfaces were analyzed by a FACSCalibur (Becton Dickinson Immunocytometry Systems, San Jose, CA, USA). Dead cells and debris were excluded from the analysis by forward- and side-scatter gating.

### Statistical analysis

Statistical differences compared to the value of negative control (NC) were evaluated using one-way ANOVA (Dunnett’s test). A probability of *p* < 0.05 was considered statistically significant. For graphical representation of data, y-axis error bars indicate the standard error of mean (SE) of the data for each point on the graph.

## Additional Information

**How to cite this article**: Endo, S. *et al.* TEX101, a glycoprotein essential for sperm fertility, is required for stable expression of Ly6k on testicular germ cells. *Sci. Rep.* **6**, 23616; doi: 10.1038/srep23616 (2016).

## Supplementary Material

Supplementary Information
