# Supplementary material for: TEX101, a glycoprotein essential for sperm fertility, is required for stable expression of Ly6k on testicular germ cells
Source: Sci Rep. 2016 Mar 23;6:23616. doi: 10.1038/srep23616 (PMC4804279; doi:10.1038/srep23616)
Supplement: Supplementary Information [file srep23616-s1.doc]

**TEX101, a glycoprotein essential for sperm fertility, is required for stable expression of Ly6k on testicular germ cells.**

Shuichiro Endo, Hiroshi Yoshitake, Hiroki Tsukamoto, Hideyuki Matsuura, Ko Kato,

Mayumi Sakuraba, Kenji Takamori, Hiroshi Fujiwara, Satoru Takeda & Yoshihiko Araki

**Supplementary Table S1** Expression *Tex101* and its related genes in *Tex101^-/-^* and *Tex101^+/+^*mice testes analyzed by microarray

ID Symbol Description Expression ratio^a^

(*Tex101^-/-^*/*Tex101^+/+^*)

M200004616 *Tex101* testis expressed gene 101 [MGI:1930791] 0.44

M200014514 *Ly6k* lymphocyte antigen 6 complex, locus K [MGI:1923736] 1.24

M300007969 *Dpep3* dipeptidase 3 [MGI:1919104] 1.04

M300002327 *Ace* angiotensin I converting enzyme [MGI:87874] 1.26

M200002190  *Adam3* a disintegrin and metallopeptidase domain 3 [MGI:102518] 0.89

M400007923 *Spaca4* sperm acrosome associated 4 [MGI:1916613] 1.04

^a^The data shown are averages of three individual experiments.

**Supplementary Table S2** Primer sets used for qRT-PCR.

Gene Forward Reverse

*Tex101* 5’-CAGGTCTTGATCGGCTCTTC-3’ 5’-GCAAAGTTCTCCTGGATTGC-3’

*Tex101** 5’-GCCCTGTGCCAATGGCACAAC-3’ 5’-ATCAGTCTGCAGCCGATCGTAG-3’

*Ly6k* 5’-ACAGCTATGCCTGCTCGAAT-3’ 5’-CCTTAGGCTCAGATGGTGGA-3’

*Adam3* 5’-GTCCATGATGGCACTGTTTG-3’ 5’-CTCTGGAGGAGCAAAACCAG-3’

*Dpep3* 5’-GAGCTTCAAGGTGTCCTTCG-3’ 5’-TGAGCTCTCTGGAGGATGT-3’

*β-actin* 5’-CATCCGTAAAGACCTCTATGCCAAC-3’ 5’-ATGGAGCCACCGATCCACA-3’

*Gapdh* 5’-TGATGACATCAAGAAGGTGGTGAAG-3’ 5’-TCCTTGGAGGCCATGTAGGCCAT-3’

*r-luc* 5’-GGATTCTTTTCCAATGCTATTGTT-3’ 5’-AAGACCTTTTACTTTGACAAATTCAGT-3’

*Rps18* 5’-TTTGCGAGTACTCAACACCAACATC-3’ 5’-GAGCATATCTTCGGCCCACAC-3’

*: Primer set for *Tex101*-transfected HEK293κB cells
